# Mapping GPs’ motivation — it’s not all about the money: a nationwide cross-sectional survey study from Denmark

**DOI:** 10.3399/BJGP.2022.0563

**Published:** 2023-08-08

**Authors:** Dimitar Yordanov, Anne Sophie Oxholm, Dorte Gyrd-Hansen, Line Bjørnskov Pedersen

**Affiliations:** Danish Centre for Health Economics, University of Southern Denmark;; Danish Centre for Health Economics, University of Southern Denmark;; Danish Centre for Health Economics, University of Southern Denmark;; Danish Centre for Health Economics and Research Unit for General Practice, University of Southern Denmark, Odense.

**Keywords:** Denmark, general practice, personnel retention, prosocial motivation, quality of care, self-centred motivation

## Abstract

**Background:**

Understanding physicians’ motivation may be essential for policymakers if they are to design policies that cater to physicians’ wellbeing, job retention, and quality of care. However, physicians’ motivation remains an understudied area.

**Aim:**

To map GPs’ work motivation.

**Design and setting:**

A cross-sectional analysis using registry and survey data from Denmark.

**Method:**

Survey data were used to measure four types of motivation: extrinsic motivation, intrinsic motivation, user orientation, and public service motivation. These were combined with register data on the characteristics of the GP, practice, and area. Using latent profile analysis, the heterogeneity in GPs’ motivation was explored; the associations between GPs’ motivation and the GP, practice, and area characteristics were estimated using linear regression analyses.

**Results:**

There was substantial heterogeneity in GPs’ motivations. Five classes of GPs were identified with different work motivations: class 1 ‘it is less about the money’ — probability of class membership 53.2%; class 2 ‘it is about everything’ — 26.5%; class 3 ‘it is about helping others’ — 8.6%; class 4 ‘it is about the work’ — 8.2%; and class 5 ‘it is about the money and the patient’ — 3.5%. Linear regression analyses showed that motivation was associated with GP, practice, and area characteristics to a limited extent only.

**Conclusion:**

GPs differ in their work motivations. The finding that, for many GPs, ‘it is not all about the money’ indicated that their different motivations should be considered when designing new policies and organisational structures to retain the workforce and ensure a high quality of care.

## INTRODUCTION

Worldwide there is a physician shortage,^1^^–^3 and physicians’ wellbeing is being challenged.^4^^–^6 Literature reviews show that physicians’ wellbeing is associated with retention^7^^–^10 and quality of care;^11^^,^^12^ as such, a key to fostering physicians’ wellbeing may be to understand their work motivations. Literature outside of healthcare acknowledges that motivation is important for performance,^13^^–^17 but physicians’ motivation remains an understudied area. This study aims to map the motivation of GPs in the Danish setting.

Four key dimensions of motivation, which may influence GPs’ wellbeing and behaviour,^18^^–^22 are:

- extrinsic motivation;
- intrinsic motivation;
- user orientation; and
- public service motivation.

Extrinsic and intrinsic motivations are so-called self-centred motivations. Individuals who are extrinsically motivated engage in activities because of the presence of tangible incentives,^20^ whereas those who are intrinsically motivated engage in activities because of a genuine interest in, and enjoyment of, the work.^23^ User orientation and public service motivation are prosocial motivations reflecting a wish to exert effort to benefit others,^22^^,^^24^^,^^25^ which relates to the concept of altruism.^25^^,^^26^ Individuals who are user oriented deliver services with the purpose of doing good for specific others (for example, patients),^21^ whereas those who are motivated by public service deliver services in order to do good for society.^27^

Agency theory^18^^,^^28^^,^^29^ represents a theoretical justification for focusing on GPs’ self-centred and prosocial motivations. The theory shows trade-offs between the agents’ (GPs’) self-centred interests (extrinsic and intrinsic motivations) and altruistic concerns (user orientation and public service motivations) towards their principals (patients and society). According to this theory, if policymakers are to ensure the wellbeing of GPs and design policies that generate the intended responses, it is important to know whether GPs are primarily incentivised by: tangible rewards, such as money (extrinsic motivation); their own professional interests (intrinsic motivation); improving patients’ health benefits (user orientation); or delivering cost-effective treatments to society (public service motivation).

Empirical evidence on healthcare providers’ motivation has mainly focused on a limited range of motivational components^16^^,^^20^^,^^21^^,^^30^ and rarely on GPs.^19^^,^^26^^,^^31^^,^^32^ Sicsic *et al*^32^ found a negative relationship between extrinsic and intrinsic motivation among French GPs. Pedersen *et al*^31^ found evidence of ‘crowding in’ of intrinsic motivation among Danish GPs being accredited. Pedersen *et al*^19^ found that risk of burnout, when accredited, was linked to Danish GPs with high intrinsic motivation, and Jensen and Andersen^26^ found that Danish GPs with high public service motivation prescribed fewer broad-spectrum antibiotics, while GPs with high user orientation prescribed more antibiotics. Supplementary Box S1 describes how the motivational components have been used in the broader literature. Although limited, the evidence indicates that motivation may be important for GPs’ wellbeing and behaviour, and can be affected by policies; however, more evidence is needed.

**Table:** How this fits in

Understanding GPs’ motivation remains an understudied area, but may be essential for designing policies and organisational structures that ensure GP wellbeing and retention, along with high-quality care. This study found heterogeneity in GPs’ work motivation and identified five GP segments; the largest comprised GPs who were motivated ‘less by the money’.

The study presented here aimed to contribute to the literature in several ways:

- by uncovering heterogeneity in GPs’ motivation using descriptive statistics, and the interdependence of different types of motivation using Pearson’s correlation coefficients;
- by identifying segments of GPs based on their motivation using latent profile analysis; and
- by estimating the associations between GPs’ motivations and GP, practice, and area characteristics using linear regression analyses.

Knowledge about the heterogeneity of motivations (including the segments of GPs) can guide how motivations should be considered when designing policies to retain GPs and ensure quality of care. For example, GPs who are more motivated by public service may be more responsive to guidelines, whereas those who are more extrinsically motivated may respond more to tangible incentives, such as bonuses; GPs who are more user oriented may be more dissatisfied if work pressures negatively affect the provided quality of care, whereas GPs who are more intrinsically motivated may feel that pressure deters professional curiosity.^33^^,^^34^ Knowledge about the interdependence of the motivations could also help to reveal whether policies need to target each motivation separately. Information about the associations between GP motivation and observable characteristics could give an insight into potentials for targeting specific groups’ motivation when designing policies.

## METHOD

### Institutional setting

In 2019, approximately 3350 GPs were registered in 1720 single-handed or partnership practices in Denmark.^35^ GPs are self-employed and work under contract with the Danish administrative regions. One-third of their income comes from capitation and two-thirds from fees for services (there is no pay for performance);^36^ these payments are the GPs’ main tangible incentives. The institutional setting supports the importance of studying the selected key motivational components.

### Data

Data were taken from the 2019 Danish national GP work–life survey, the primary objective of which was to collect information on motivation. The survey includes items measuring extrinsic motivation, intrinsic motivation, user orientation, and public service motivation (Supplementary Table S1). All 3336 privately practising GPs who were registered with a practice provider number in the Danish Health Authorities’ Organisation Register at the start of 2019 received an invitation to participate in the survey. For this study, the GPs’ authorisation identification numbers and postal codes were used to link survey data on motivation to high-quality register data on GP, practice, and area characteristics; further details are given in Supplementary Box S2.

### Empirical approach

#### Constructing simple sum scores for the motivational components

Confirmatory factor analyses were used to investigate how each survey item contributed to the latent constructs of the four components. In line with Pedersen *et al*’s^31^ methodology, a single sum score was constructed for each component. For each item, a five-point Likert scale was converted to a numeric scale by being assigned a number from one (‘completely disagree’) to five (‘completely agree’), with the numbers within each motivational component then added together. The scores were standardised to range from zero to 100 using the minimum–maximum approach;^37^ zero indicated the lowest observed value (the least motivated GPs), and 100 indicated the highest observed value (the most motivated GPs) for each motivational component (for further details see Supplementary Tables S2‒S5).

#### Exploring heterogeneity in GPs’ motivation

Descriptive statistics were presented using a violin plot to explore variation in the motivational components. Comparisons across components were not conducted, as the components were measured using different items.

#### Exploring interdependence between motivational components

Pearson’s correlation coefficients between the four motivational components were estimated to investigate their interdependence.

#### Identifying segments of GPs

Latent profile analysis was used to identify segments of GPs based on their motivations. To identify which motivational components were predominant in each class, the coefficients in each class were compared against the overall sample mean within each component and tested to ascertain whether they were statistically significantly different; a specification of the model is given in Supplementary Box S3.

#### Associations between motivation and GP, practice, and area characteristics

Ordinary least squares regression models, in which standard errors were clustered at practice level, were used to investigate whether GP motivation was associated with GP, practice, and area characteristics. Motivation was measured as:

- GPs’ probability of class membership for each class identified in the latent profile analysis; and
- GPs’ score on each motivational component, while controlling for other motivational components. (Specifications of the models are given in Supplementary Box S4).

Details of the supplementary analyses are given in Supplementary Tables S11– S16.

## RESULTS

A total of 1152 GPs completed the survey, giving a response rate of 34.5%. The responding GPs were, to a large extent, representative of the GP population in Denmark. Responders’ practices were mainly located in the Region of Southern Denmark and the Central Denmark Region (Supplementary Table S6).

### Exploring heterogeneity in GPs’ motivation

**Figure 1.**
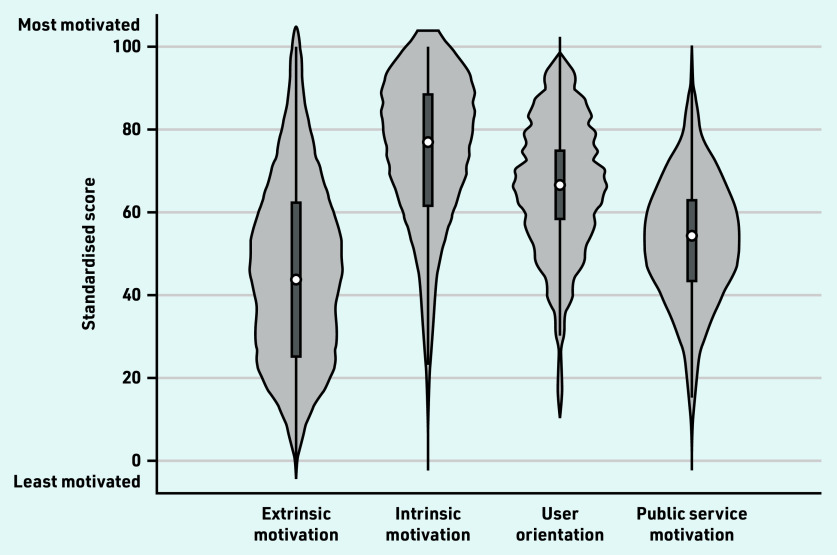
*Violin plot showing variation in GPs’ standardised simple sum scores across motivational components.^a^* *^a^The circle indicates the median, the black box indicates the interquartile range, spikes indicate the upper and lower adjacent values, and the shaded area indicates the kernel density distribution.*

Figure 1 illustrates the heterogeneity of the motivational components; standard deviations ranged between 15 and 23 (Supplementary Table S7). Extrinsic motivation, user orientation, and public service motivation were distributed fairly symmetrically, with user orientation and public service motivation approximately following an even distribution. Intrinsic motivation was skewed to the left (most GPs having intrinsic motivation above the average), while extrinsic motivation had a bimodal distribution, indicating that there were two groups of GPs with different levels of extrinsic motivation.

### Exploring interdependence between motivational components

Table 1 shows that there was a low correlation between the motivational components (defined as below ±0.3, in line with the work of Hinkle *et al*).^38^ This result indicates that the components did not illustrate the same type of motivation.

**Table 1.** Pearson’s correlation coefficients between the four motivational components

	**Extrinsic motivation (*P*-value)**	**Intrinsic motivation (*P*-value)**	**User orientation (*P*-value)**	**Public service motivation (*P*-value)**
Extrinsic motivation (*P*-value)	1.000			
Intrinsic motivation (*P*-value)	−0.037 (0.213)	1.000		
User orientation (*P*-value)	0.116^a^ (<0.001)	0.039 (0.191)	1.000	
Public service motivation (*P*-value)	−0.059^a^ (0.044)	0.166^a^ (<0.001)	0.119^a^ (<0.001)	1.000

a *Statistically significant (*P<*0.05)*.

### Identifying segments of GPs

**Figure 2.**
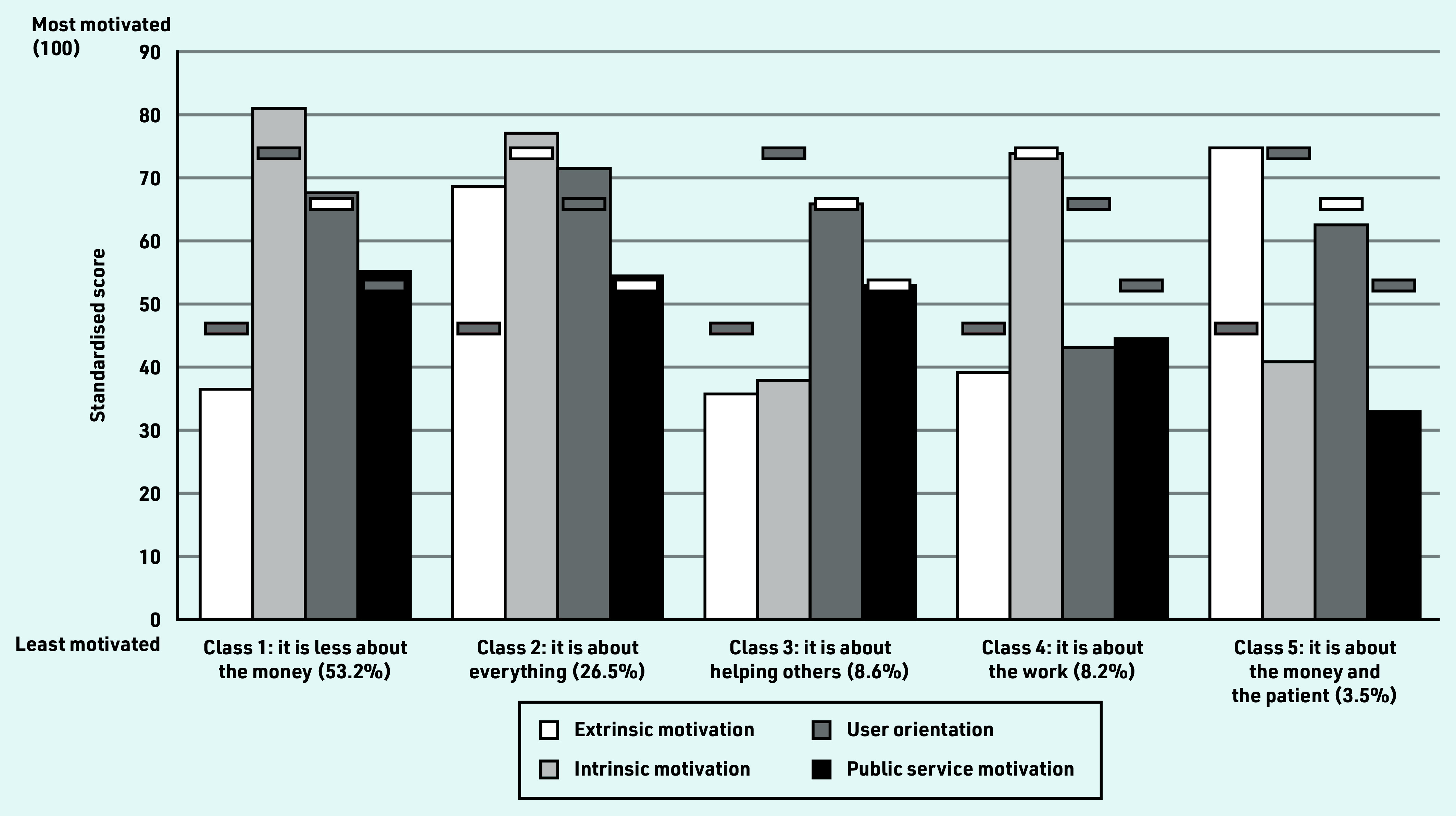
*Class composition and mean standardised simple sum scores of the four motivational components across the five classes.^a^* *^a^The five classes are listed with probability of class membership in parentheses. The horizontal bars indicate the mean score for all GPs for each of the motivational components. The horizontal bars are shaded if there is a statistically significant (*P*<0.05) difference between the mean score for all GPs and the mean class score (tested using an unequal variance*t*-test).*

Figure 2 illustrates the results from the latent profile analysis (Supplementary Table S8 presents the estimates). Based on the fit statistics (Supplementary Table S9) and interpretability of the classes, the five-class model was chosen:

- class 1 (probability of class membership 53.2%) ‘it is less about the money’ was characterised by intrinsic motivation, user orientation, and public service motivation being at or above the GP mean, while extrinsic motivation was statistically significantly below the mean;
- class 2 (26.5%) ‘it is about everything’ was characterised by all motivations being at or above the GP mean;
- class 3 (8.6%) ‘it is about helping others’ was characterised by extrinsic motivation and intrinsic motivation being statistically significantly below the mean, and user orientation and public service motivation being at the mean;
- class 4 (8.2%) ‘it is about the work’ was characterised by extrinsic motivation, user orientation, and public service motivation being statistically significantly below the mean, and intrinsic motivation being at the mean level; and
- class 5 (3.5%) ‘it is about the money and the patient’ was characterised by intrinsic motivation and public service motivation being statistically significantly below the mean, and extrinsic motivation and user orientation being at or above the mean.

### Associations between GPs’ motivation and GP, practice, and area characteristics

Supplementary Table S10 shows the associations between GP, practice, and area characteristics and individual probability of class membership or GPs’ motivational scores. The observable characteristics are associated with GP motivation only to a limited extent; specifically, they explain between 0.3% and 3.1% of the variation in the individual probability of class membership and between 3.5% and 5.7% of the variation in the motivational scores. Male GPs seem to be more extrinsically and prosocially motivated compared with female GPs, who are more intrinsically motivated. Younger GPs tend to be less prosocially motivated than older GPs. The results from the supplementary analyses support those of the main analysis (Supplementary Tables S11–S16).

## DISCUSSION

### Summary

Heterogeneity in GPs’ motivation was found in all motivational components. Interestingly, the distribution of extrinsic motivation was bimodal, suggesting that there were two groups of GPs in the sample for whom tangible incentives were not equally important. The authors also found that the four motivational components were only weakly correlated with each other. Five classes of GPs with different motivational profiles were identified. Class 1 ‘it is less about the money’ (membership of class probability: 53.2%), class 3 ‘it is about helping others’ (8.6%), and class 4 ‘it is about the work’ (8.2%) were characterised by being less extrinsically motivated relative to the mean. GP, practice, and area characteristics were only associated with motivation to a limited extent.

### Strengths and limitations

To the best of the authors’ knowledge, this study was the first to map physicians’ motivation using four components that reflect complementary key areas of motivation. Self-reported measures of motivation were utilised and combined with high-quality register data on GP, practice, and area characteristics. The results may be generalisable to GPs practising in high-income countries with systems that primarily follow the Beveridge model (for example, England and Norway). However, as this study is the first to comprehensively map physicians’ motivation, more research is needed to verify the generalisability of the findings; as an example, it would be useful to ascertain whether the findings apply to GPs in systems that follow other types of healthcare models, to GPs in low- and middle-income countries, and to other types of healthcare providers. More knowledge is also needed about whether motivational profiles are stable across time and contexts.

It is impossible to say that classic biases, such as self-selection bias, social desirability bias, or strategic bias,^39^^–^41 were not present in the study presented here; however, the authors believe that the problem is minimal. A large proportion of the GPs responded, which reduces the risk of self-selection bias. Responses were provided anonymously, which reduces risk of social desirability bias. Heterogeneous motivation was found across GPs, and also in extrinsic motivation, where social desirability bias might be most pronounced. Finally, the authors tried to minimise the risk of strategic bias by posting neutral questions in the survey.

### Comparison with existing literature

The motivational components were only weakly correlated with each other. This finding aligns with those of Sicsic e*t al*^32^ and Dill *et al*,^30^ who studied extrinsic and intrinsic motivation among French GPs and hospital nurses in the US, respectively, and Jensen and Andersen,^26^ who studied user orientation and public service motivation among Danish GPs. A systematic review by Marchand and Peckham^3^ showed that tangible incentives, such as money, were less important than other motivational factors for GP recruitment and retention; this result is aligned with the finding presented here that more than half of GPs belonged to a segment in which extrinsic motivation was less important.

Although only a few other studies^19^^,^^26^^,^^31^ have examined GPs’ motivation using the same motivational components as those in the study presented here, others have explored specific GP motives that could constitute dimensions under the general measures of motivation. For example, a study found that some GPs value flexibility in their work,^42^ indicating a need for autonomy, which is a typical trait among GPs with high levels of intrinsic motivation.^33^^,^^34^ Another study found that GPs engage in teaching for different reasons: some simply enjoy teaching (intrinsic motivation), some want to update their clinical knowledge to help patients (user orientation), and others consider teaching to be a responsibility they have to the community (public service motivation).^43^ These studies^42^^,^^43^ support the authors’ finding that GPs are heterogeneous in their work motivation.

### Implications for research and practice

Understanding GPs’ motivation could help ensure GPs’ wellbeing and solve issues with GP shortages and quality of care.^1^^–^6 If decision makers take into account differences in GPs’ motivations in their planning of general practice, they may reduce GP shortage by retaining or recruiting GPs. This may require a flexible general practice organisation, in which GPs can self-select into contracts that differ in terms of employment (for example, salaried versus privately practising), degree of patient contact, and opportunities to engage in activities for the benefit of society or their own professional interests.

Literature outside of the healthcare setting has shown that motivation is important for workers’ performance.^13^^–^17 It is, therefore, likely that the heterogeneity in GPs’ motivational profiles may explain variation in their treatment behaviour, beyond what has been shown for prescriptions of antibiotics.^26^ Such variation could lead to differences in quality of care and inequality in access to care.^44^

The low correlation between the motivational components suggests that they measure different aspects of motivation; as such, incentive schemes may be more effective if they target different types of motivations. The findings presented here may explain why other studies have found that GPs do not always respond to financial incentives.^45^^–^47 Similarly to the conclusions drawn by Lagarde *et al*,^48^ the authors suggest that GPs who are not highly extrinsically motivated may respond better to incentives targeted at their other motivations; policymakers should, therefore, consider using a mix of financial and non-financial incentives. Studies exploring how different types of incentives link to GPs’ care and their motivational profiles are warranted.

Although some statistically significant associations were found between GPs’ motivation and their age and gender, observable GP, practice, and area characteristics seemed not to be strongly associated with motivation, as the characteristics only explained a small proportion of the variation in motivation. The authors therefore suggest that GPs’ motivation is taken into consideration, in addition to these other observable characteristics, when designing policies, as observable characteristics alone seemed not to be good predictors for motivation.

## Data

The data that support the findings of this study cannot be shared because of legal restrictions.
